# hnRNP E1 at the crossroads of translational regulation of epithelial-mesenchymal transition

**DOI:** 10.20517/2394-4722.2018.85

**Published:** 2019-03-11

**Authors:** Simon Grelet, Philip H. Howe

**Affiliations:** 1 Hollings Cancer Center, Medical University of South Carolina, Charleston, South Carolina 29425, USA.; 2 Department of Biochemistry, Medical University of South Carolina, Charleston, South Carolina 29425, USA.

**Keywords:** Breast cancer, tumor progression, epithelial-mesenchymal transition, cancer stem cells, transforming growth factor-β, translation, hnRNP E1, *PCBP1*

## Abstract

The epithelial-mesenchymal transition (EMT), in which cells undergo a switch from a polarized, epithelial phenotype to a highly motile fibroblastic or mesenchymal phenotype is fundamental during embryonic development and can be reactivated in a variety of diseases including cancer. Spatio-temporally-regulated mechanisms are constantly orchestrated to allow cells to adapt to their constantly changing environments when disseminating to distant organs. Although numerous transcriptional regulatory factors are currently well-characterized, the post-transcriptional control of EMT requires continued investigation. The hnRNP E1 protein displays a major role in the control of tumor cell plasticity by regulating the translatome through multiple non-redundant mechanisms, and this role is exemplified when E1 is absent. hnRNP E1 binding to RNA molecules leads to direct or indirect translational regulation of specific sets of proteins: (1) hnRNP E1 binding to specific targets has a direct role in translation by preventing elongation of translation; (2) hnRNP E1-dependent alternative splicing can prevent the generation of a competing long non-coding RNA that acts as a decoy for microRNAs (miRNAs) involved in translational inhibition of EMT master regulators; (3) hnRNP E1 binding to the 3’ untranslated region of transcripts can also positively regulate the stability of certain mRNAs to improve their translation. Globally, hnRNP E1 appears to control proteome reprogramming during cell plasticity, either by direct or indirect regulation of protein translation.

## INTRODUCTION

### Epithelial-mesenchymal transition in tumor progression and metastasis

Metastasis represents a critical step in tumor progression and accounts for more than 90% of cancer-induced mortality^[1]^. Despite the tremendous efforts made by the scientific community over recent decades, the cellular and molecular events that control tumor cell plasticity remain incompletely elucidated.

**Figure 1.**
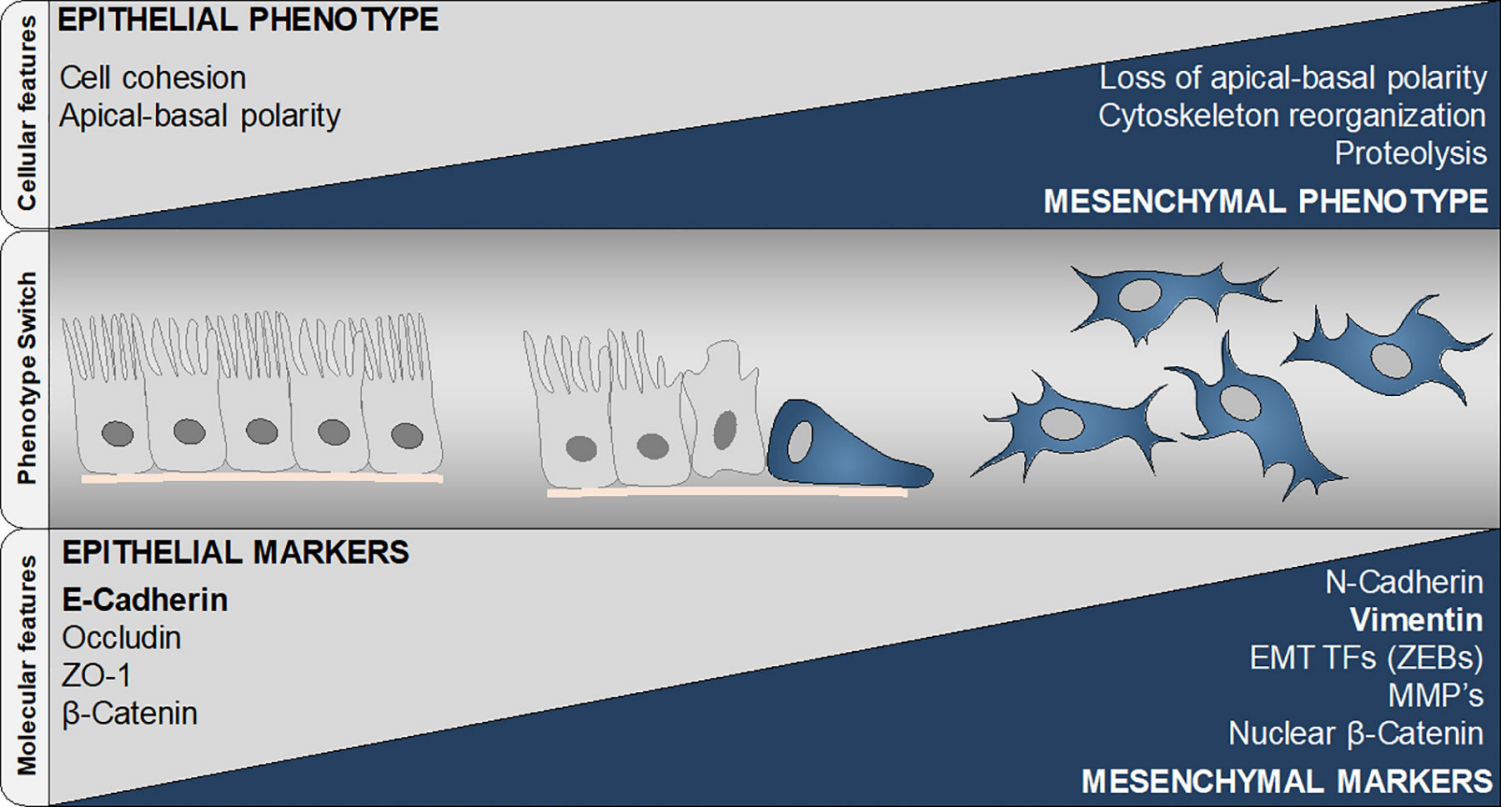
Epithelial-mesenchymal transition relies upon a gradually orchestrated switch from a polarized, epithelial phenotype to a highly motile fibroblastic or mesenchymal phenotype. Epithelial cells are polarized with strong cell-cell cohesions and are organized by multiple cell junction proteins such as E-Cadherin, Occludin, Zonula Occludens, β-catenin and other epithelial markers. During EMT, tumor cells lose their epithelial features and acquire a mesenchymal phenotype, which promotes their motility and invasive capacity. The switch is acquired through a deep reprogramming of the transcriptional landscape and involves activation of EMT transcription factors such as ZEBs, reorganization of cytoskeletal components by regulation of proteins such as Vimentin, and modulation of expression/secretion of invasion-mediating proteases such as matrix metalloproteinases

It has been shown that the epithelial-mesenchymal transition (EMT) is essential in embryonic development and in tumor metastasis and is among the mechanisms deemed critical in tumor cell plasticity. EMT consists of a fine-tuned phenotypic switch, characterized by the loss of apical-basal polarity and cellular adhesion in epithelial cells^[2,3]^. Cells undergoing transition gradually express mesenchymal features, such as enhanced cytoskeletal rearrangement and extracellular matrix (ECM) degradation, both essential for cell motility [Figure 1].

EMT is not a unidirectional mechanism. The transition is a fine-tuned, reversible mechanism that allows cells to switch between epithelial and mesenchymal phenotypes while manifesting all intermediate phenotypic shades^[2,4]^. The reverse mechanism, known as mesenchymal-epithelial transition (MET), allows reversion to the differentiated phenotype. Reversion is important for the potential formation of metastases that can occur as tumor cells attempt to relocate to distant organs to develop secondary tumors. Due to its transience, its presence in multiple-states, and its reversible nature, EMT is technically challenging to observe throughout tumor progression *in vivo*^[4]^. Nevertheless, it is clearly demonstrated that transitioned cells harbor higher invasive capacities^[5–7]^. In the early steps of metastasis, epithelial cancer cells must acquire the ability to separate from the primary tumor^[4,8]^. Such departure may occur as single cells or as clusters of cells, and always requires the loss or the alteration of cell-to-cell and cell-to-matrix interactions^[9]^.

In the current model, EMT-positive tumor cells displaying newly-acquired mesenchymal features invade their surrounding environment and intravasate into the circulatory system. Cancer dissemination in this model results from ECM degradation and increased motility^[10,11]^. It was also proposed that the survival of circulating tumor cells (CTCs) in the blood stream was enhanced by the phenotypic plasticity observed during EMT^[12–15]^. Following dissemination into the circulation, CTCs extravasate and colonize distant organs to ultimately form secondary tumors. The ultimate metastatic colonization occurs through the reepithelialization of cells by MET and is followed either by a proliferative cycle with subsequent drug resistant secondary tumor growth, or by a dormant cycle with latency of tumor relapse. Although there are countless reports demonstrating that genetic mutations are recurrently arising in many types of primary tumors, the attempts to identify genes that are recurrently mutated in the genomes of metastasized cells have consistently failed until now. Such observations are advocating for the prime role of cell plasticity in tumor cells dissemination. However, the experimental evidences of the casual function of EMT in metastasis formation remain to be clearly demonstrated. The explicit role of EMT in tumor progression remains actively debated but its implication in the increased resistance seen in both conventional and targeted antitumor therapies is currently well-accepted by the scientific community^[12,14,16–21]^. I seems now clear that the controversy around the role of EMT in tumor dissemination might be attributed to its non-linear and multi-modal nature and that these many intermediate stages of EMT may occupy different regions on such a multi-dimensional landscape^[22]^. Many groups are still investigating the role of EMT in tumor cells dissemination, and at this point, multiple hypotheses are still emerging to underline the mechanisms involved in the successful dissemination of tumor cells beyond the current EMT/MET view^[23]^.

### Heterogeneous nuclear ribonucleoprotein E1

Heterogeneous nuclear ribonucleoproteins (hnRNPs) encompass a large family of RNA-binding proteins (RBPs) that contribute to multiple aspects of nucleic acid metabolism. These aspects include alternative splicing, mRNA stabilization, transcriptional control, and translation regulation. The coding sequences of hnRNPs reveal a modular structure consisting of one or more RNA-binding motifs, and at least one auxiliary domain that regulates protein-protein interactions and subcellular localization^[24]^. Indeed, all hnRNPs contain RNA binding domains that may include RNA recognition motifs (RRM), the quasi-RRM, glycine-rich domains and KH domains. Contrary to RNA-binding domains, auxiliary domains are divergent in protein sequence and are unstructured but are highly involved in regulating subcellular localization and other biological features. Most of the hnRNP proteins contain nuclear localization signals and are therefore mainly located in the nucleus during steady state. However, they can translocate into the cytosol via signaling pathway activation or by recruitment by other proteins. Importantly, for most of the hnRNPs, cellular functions are tightly regulated through post-transcriptional modifications including but not limited to methylation, phosphorylation, ubiquitination and sumoylation.

Poly(rC)-binding protein 1 (PCBP1 also called hnRNP E1) belongs to a hnRNP family that is composed of hnRNP K/J and the alpha-complex proteins (PCBP1–4α or CP1–4). Proteins in this family contain three hnRNP K homology (KH) domains for RNA-binding. The human PCBP1 gene encodes the hnRNP E1 protein, and was initially defined as clone sub2.3, with poly(C)-binding activity and observed similarity to hnRNP E2^[25]^. hnRNP E1 harbors three highly conserved KH domains, KH1 to KH3. KH1 and KH3 domains were initially predicted to bind to RNA and *in vitro* studies later showed that the KH2 domain of hnRNP E1 also binds to RNA to regulate protein translation^[26–28]^. The hnRNP E1 and hnRNP E2 proteins share 82% amino acid identity, with an even higher level of conservation (93%) for their KH domains^[24]^.

The subcellular localization of hnRNP E1 is predominantly nuclear, and demonstrates accumulation within nuclear speckles, with precise sites of accumulation observed with splicing factors prior to nascent transcript loading^[29]^. The nuclear localization of hnRNP E1 is abolished by Actinomycin D^[30]^. Since splicing factors undergo continuous and rapid nucleo-cytoplasmic shuttling^[31]^, and because splicing is coupled to transcription, RNA polymerase inhibition results in the cytoplasmic accumulation of many splicing factors. Such observations support the primordial nuclear role of hnRNP E1 in pre-RNA splicing^[32]^. The subcellular localization of hnRNP E1 is also governed by cell signaling stimuli. It was demonstrated that SMAD3 and hnRNP E1 were induced to govern alternative splicing, mediated by their colocalization in SC35 (also known as SRSF2)-positive nuclear speckles, downstream of EGF and TGFb, respectively^[33]^. Even if it is to a lesser extent, hnRNP E1 is also clearly observed in the cytoplasm, and its presence potentially correlates to its function as a translational regulator^[29,34,35]^.

## REGULATION OF TRANSCRIPTION

HnRNP E1 functions as a regulator of gene expression at the transcriptional level, although this does not appear to be its primary role. Recombinant cloning of the four members (PCBP1 to PCBP4) of the poly(C) binding protein family (PCBP) demonstrated that transcriptional activity of the mouse μ-opioid receptor (MOR) gene increased due to interaction between PCBPs and the 26-bp polypyrimidine stretch in the MOR proximal promoter^[36]^. PCBPs can bind either to single-stranded or to double-stranded DNA^[27]^. hnRNP E1 also exhibits a mild but consistent activation of the promoter of the BRCA1 gene^[37]^. Finally, hnRNP E1 was found to regulate eIF4E transcription^[38]^. Recruitment of hnRNP E1 to the eIF4E basal element (4EBE) in the eIF4E promoter region occurs downstream of serum or EGF-mediated cellular stimulation, and this mechanism requires Pak1-dependent phosphorylation of hnRNP E1 protein^[38]^. These findings suggest that both hnRNP E1 and its phosphorylation downstream of growth factor-induced signaling play a regulatory role in eIF4E transcription in mitogen-stimulated cells.

## TRANSLATION REGULATION

The hnRNP E1 protein regulates translation through either direct or indirect mechanisms. Examples of the most common mechanisms include regulation of mRNA stability, direct control of the ribosomal machinery, or the generation of RNA species that prevent miRNA-mediated translational repression of specific mRNAs.

### Control of mRNA stability

The role of hnRNP E1 in RNA stability is exemplified by a broad spectrum of mRNA interactions. Mainly, hnRNP E1 regulates gene expression via binding to specific AU-rich elements (AREs) or U-rich elements located in the 3’ untranslated regions (UTR) of target mRNAs. For instance, the binding of hnRNP E1 to p27^kip1^ 3’ UTR via its KH1 domain stabilizes p27^kip1^ mRNA, fueling p27^kip1^ protein expression by enhancing its translation prior to degradation. The upregulated p27^kip1^ protein consequently inhibits cell proliferation, cell cycle progression, and tumorigenesis, and can concurrently promote cell apoptosis under paclitaxel treatment^[39]^. Interestingly, other cyclin-dependent kinase inhibitors such as p21^Waf1^ are also regulated through hnRNP E1 mediated mRNA stability. Co-immunopurification of p21^Waf1^ mRNA from MDAMB-468 breast cancer cells using hnRNP E1 antibody suggests that hnRNP E1 protein binds to one or more motifs distributed throughout the p21^Waf1^ 3’ UTR to stabilize the mRNA^[30]^.

The eNOS mRNA 3’ UTR contains multiple evolutionarily conserved pyrimidine (C and CU)-rich sequence elements that are both necessary and sufficient for mRNA stabilization. The hnRNP E1 protein binds to these 3’ UTR elements. Hence, hnRNP E1is recruited to a stabilizing RNP complex that protects eNOS mRNA from the inhibitory effects of its antisense transcript sONE, and from 3’ UTR-targeting small interfering RNAs (siRNAs) and miRNAs^[40]^. HnRNP E1 regulates the stability of p63 mRNA via binding to a CU-rich element (CUE) within the p63 3’ UTR^[41]^. The p63 protein contribution to EMT could vary according to the biological context. It has been shown that p63 directly modulates the tumor cell plasticity by either attenuating EMT in human prostate cancer cells or promoting tumor cell invasion during human and mouse breast tumor cells dissemination^[42,43]^. Phosphorylation of hnRNP E1 also contributes to stabilization of mu opioid receptor (MOR) mRNA via interaction with ARE RNA-binding protein 1 (AUF1) and poly A binding protein (PABP)^[44]^.

### Alternative polyadenylation

The addition of the poly-(A) to the messenger RNA 3’ UTR is a co-transcriptional process occurring in the nucleus. As 3’ UTRs contain cis elements that are involved in various aspects of mRNA metabolism, 3’ UTR alternative polyadenylation (APA) can affect post-transcriptional gene regulation considerably in various ways, including modulation of stability, translation, nuclear export and cellular localization of mRNA. 3’ UTR-APA can also affect the localization of the encoded protein^[45]^. Polyadenylation of mRNA is a two-step process consisting of endonucleolytic cleavage and addition of an untemplated poly(A) tail.

The role for hnRNP E1 in the regulation of alternative polyadenylation has been established in an *in vitro-*transcribed and polyadenylated alpha-globin 3’ UTR assay^[46]^. Furthermore, a screening study for alternative polyadenylation utilizing RNA interference (RNAi) identified hnRNP E1 as the second highest factor in the control of polyadenylation signal usage. The mechanism by which hnRNP E1 modulates polyadenylation has yet to be characterized^[47]^.

### Direct control of translation machinery through BAT elements

As we and others have previously demonstrated, the regulation of gene expression at the post-transcriptional level plays an indispensable role in TGFβ-induced EMT and metastasis^[30,34,48–52]^. We identified a transcript-selective translational regulatory pathway in which a ribonucleoprotein (mRNP) complex binds to a 33-nucleotide TGFβ-activated translation (BAT) element in the mRNA 3’ UTR and silences the translation of a cohort of mesenchymal protein-encoding mRNAs. HnRNP E1 is a critical component of the BAT-binding mRNP complex^[53]^. Silenced mRNAs include Disabled2 (Dab2) and Interleukin-like EMT-inducer (ILEI), which are involved in mediating EMT^[34,51,54,55]^. Furthermore, TGFβ activates a kinase cascade terminating in the phosphorylation of hnRNP E1 protein at Serine 43. This phosphorylation occurs by isoform-2 specific activation of Akt and induces the release of the mRNP complex from the BAT element. This results in the reversal of translational silencing of mesenchymal protein-encoding transcripts that are required for EMT. By using a genome-wide combinatorial approach involving expression, polysome profiling and RIP-Chip analysis, we have identified the members of the cohort of translationally regulated mRNAs that are induced during TGFβ-mediated EMT^[53]^.

**Figure 2.**
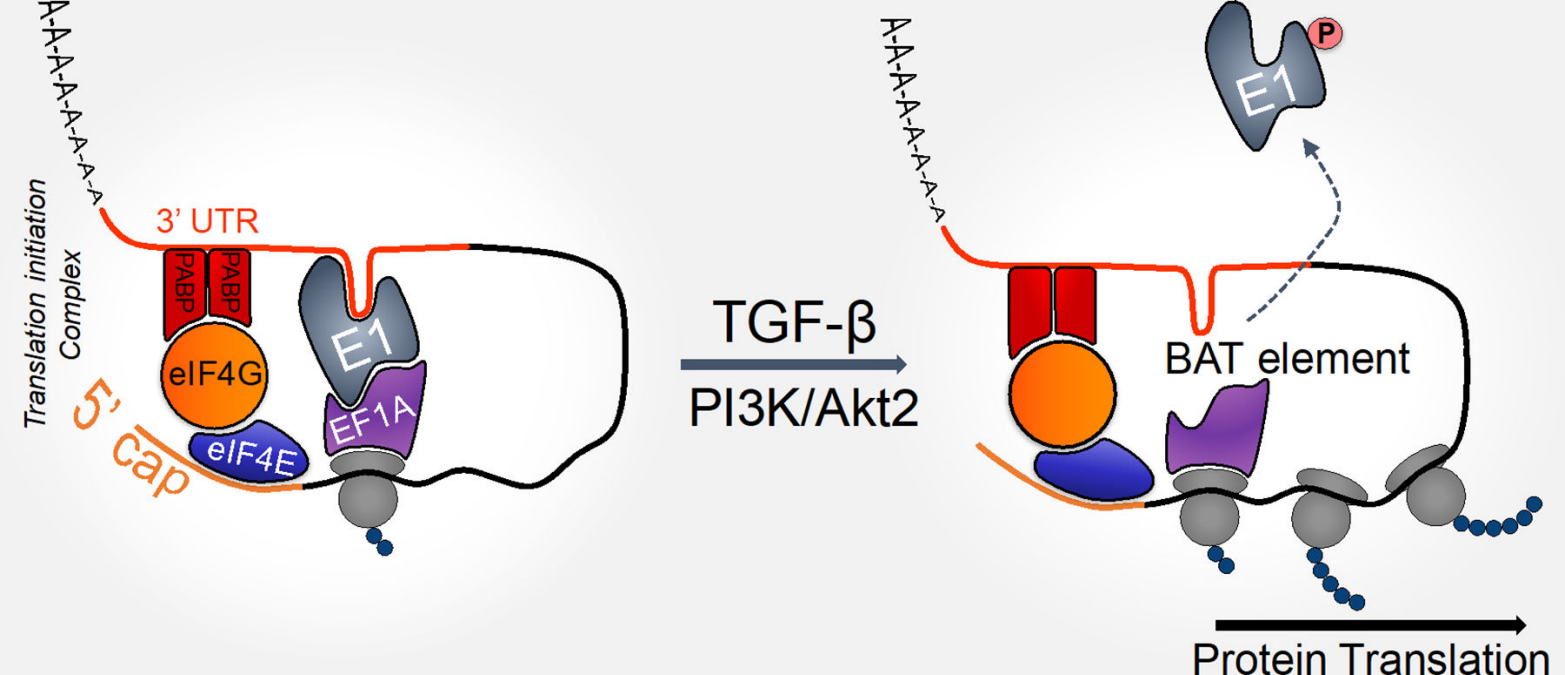
Molecular mechanism of hnRNP E1-mediated translational silencing. The eukaryotic elongation factor-1A1 (eEF1A1) forms a complex with hnRNP E1 and the BAT element, and silences specific protein expression by stalling the elongation of their translation by ribosomes. Given the necessity for cognate-codon interaction with the ribosomal A site, it is likely that the formation of the BAT mRNP complex occurs post-delivery of the aminoacyl-tRNA to the ribosome. The ability of the BAT mRNP complex to inhibit eEF1A1-dependent elongation suggests that the 3’-UTR is interacting with the 5’-UTR in a circularized model to facilitate its proximity to the 80S ribosome^[35]^. It has been suggested that translatable mRNAs are likely to be found in circular forms due to interaction between PABP, eIF4G, and the cap-binding protein eIF4E^[56]^

At the molecular level, the eukaryotic elongation factor-1 A1 (eEF1A1) is an important additional functional component of the mRNP complex. We have previously demonstrated that the BAT element, hnRNP E1 and eEF1A1 form a ternary complex that mediates translational silencing at the translational elongation step^[35]^. In non-stimulated cells exhibiting epithelial characteristics, hnRNP E1 binds to eEF1A1 and blocks progression of the 80S ribosome by preventing the release of eEF1A1 from the ribosomal A-site following GTP hydrolysis. EMT induced by either TGFβ or hnRNP E1 knockdown disrupts the mRNP complex, allowing eEF1A1-mediated translational elongation of mesenchymal transcripts to proceed^[34,35]^ [Figure 2].

This mode of translational regulation represents an unusual case of dependency upon either agonists or stimuli to upregulate translation through 3’-UTR elements. Thus, the elucidation of this post-transcriptional regulatory pathway identified an “EMT gene signature” and provided mechanistic information as to how cell plasticity could be tightly regulated. Taken together, this work underscores the contribution of the nonphosphorylated hnRNP E1 protein to maintenance of epithelial cell integrity under normal conditions. During tumor-related epithelial plasticity, hnRNP E1 also acts as the trigger for the reversal of translational silencing, resulting in a fine-tuned, spatio-temporally controlled increase in mesenchymal protein expression.

### Indirect control of translation through alternative splicing

**Figure 3.**
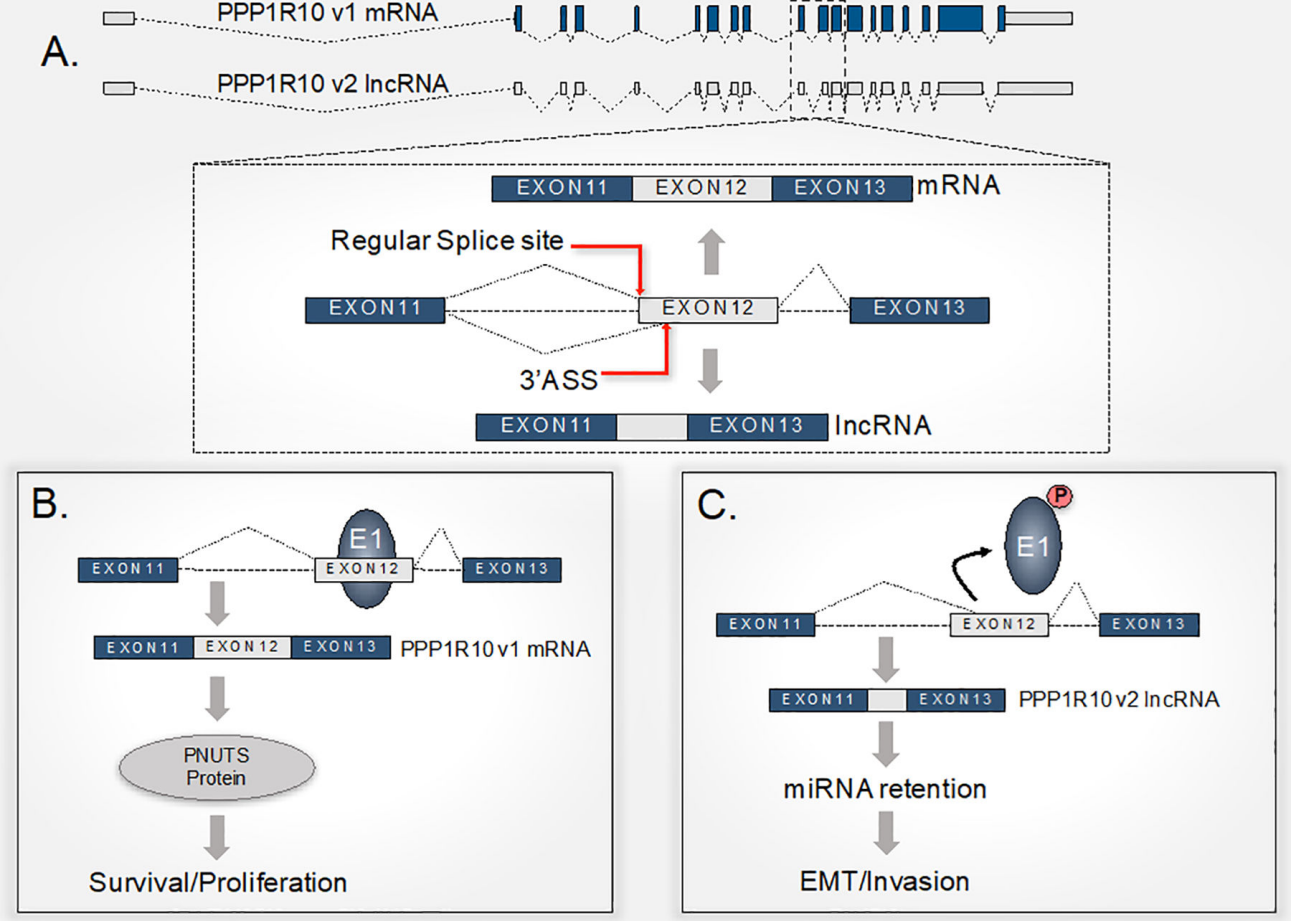
Molecular mechanism of hnRNP E1-mediated alternative splicing of *PNUTS*. A: The PPP1R10 (*PNUTS*) gene locus can encode either a protein coding mRNA or a non-coding RNA isoform. The *PNUTS* gene locus is highly conserved between human and mouse and expresses both coding and non-coding transcripts. The lncRNA-PNUTS is generated by the usage of the 3’ alternative splice site (3’ASS) located at the 5’ end of exon 12. This usage leads to the change of the open reading frame and the generation of a premature stop codon; B: the binding of hnRNP E1 to a BAT consensus element located in the alternative splicing site results on its masking and prevents its usage to generate the *PPP1R10* mRNA translated into the PNUTS protein; C: loss of hnRNP E1 binding to the alternative splice site uncovers it and allows its usage by the spliceosome machinery. The lncRNA-*PNUTS* acts as a decoy for miRNA-205 and thus allows the de-repression of ZEB protein translation. Reactivated expression of ZEB proteins induces the shutdown of epithelial markers such as E-Cadherin, allowing EMT to proceed

Alternative splicing regulates over 90% of multi-exon protein-coding genes in humans^[57]^. Abnormal regulation of alternative splicing often produces disease-specific protein isoforms^[58,59]^. Additionally, genome-wide analysis has identified tens of thousands of “splice variant” mRNAs that are enriched in a wide range of human diseases^[60,61]^. The hnRNP E1 protein is well documented for its repressive role in alternative splicing mechanisms as they apply to human health and disease. For instance, hnRNP E1 represses tumor cell invasion by inhibiting the alternative splicing of CD44^[62]^. It was therefore demonstrated that enforced hnRNP E1 expression inhibited CD44 variants expression in HepG2 human liver cancer cells while knockdown of endogenous hnRNP E1 induced these variants splicing^[62]^. Interestingly, another study based on *in vitro* and *in vivo* models of breast cancer progression demonstrated the switch of CD44 expression occurring from variant isoforms to the standard isoform during EMT. This isoform switch to CD44s was essential for cells to undergo EMT and was required for the formation of breast tumors that display EMT characteristics in mice^[63]^. HnRNP E1 also binds to the growth hormone receptor pseudoexon and prevents its usage to allow the expression of a functional protein^[64]^. Disruption of hnRNP E1 binding and subsequent activation of an alternative splicing event responsible for Laron syndrome was demonstrated either by hnRNP E1 knockdown or by alterations to the genomic pseudosite. We also recently reported the binding of hnRNP E1 to a pre-RNA pseudosite in the serine/threonine-protein phosphatase 1 regulatory subunit 10 (*PNUTS*) transcript^[30]^. The hnRNP E1 protein binds to a conserved BAT element that is similar in structure to those observed in the 3’-UTRs of the mesenchymal encoding mRNAs discussed above. The loss of hnRNP E1 binding to the alternative splicing site of *PNUTS* following hnRNP E1 knockdown, phosphorylation, and/or cytoplasmic translocation activates usage of the pseudosite and generates an alternatively spliced isoform of PNUTS. This alternative *PNUTS* isoform does not encode a functional protein, but rather a non-coding isoform of the gene. Functionally, the lncRNA-PNUTS acts as a decoy for miRNA-205 and binds to the miRNA causing a decrease in miRNA-205 bioavailability. This abolishes translational inhibition of mesenchymal factors such as ZEB mRNAs that would otherwise be suppressed in epithelial cells^[30]^. Since alternative splicing and generation of lncRNA-PNUTS is an early event in TGFß-mediated EMT, the lncRNA-PNUTS likely operates as a transient inhibitor of the miRNA-205 to allow for the temporal upregulation of ZEBs and subsequent regulation of downstream EMT events. Indeed ZEBs proteins are reciprocally linked in a feedback loop with the miR-200 family, each strictly controlling the expression of the other^[18,65]^. In this way, a transient, but nevertheless, strong decrease in miR-205 bioavailability, sufficient to activate the ZEB proteins, would allow for transcriptional repression of the miR-200 family or other miRNAs such as miR-183 or miR-203 thereby further stabilizing ZEB proteins and reinforcing the EMT process^[30]^. Moreover, it has been suggested that the feedback loop of miR-200/ZEB also generates hybrid phenotypes of the cells during EMT-mediated tumor cell plasticity^[66]^ [Figure 3].

## CONCLUSION

For many years, characterization of the role of RBPs in tumor biology and cell plasticity resulted in substantial progress, and the investigation of hnRNP E1 provided understanding of many facets of its molecular function in cells. The function of the PCBP1 gene encoding the hnRNP E1 protein was first demonstrated through characterization of its role as a negative regulator of alternative splicing. Since then, many additional roles have also been discovered, and most of them appear to have critical participation in the maintenance of cell phenotype integrity^[34,39,67]^. At the molecular level, the ability of hnRNP E1 to specifically bind to mRNA species often leads to a direct or indirect regulation of their translation. This occurs either by controlling processivity of ribosomal machinery, stabilizing mRNA, or locking/unlocking dormant translational controls. Since it is well established that hnRNP E1 controls cell plasticity in health and disease through multiple fine-tuned regulatory mechanisms, it will be essential to develop investigations involving novel therapeutic strategies. Moreover, targeting the KH domain of hnRNP E1 may be relevant. However, because hnRNP-E1 is pleiotropic and ubiquitous, confining therapeutic strategies to tumor cells may be challenging.

The phenotypic changes observed during cell plasticity and tumor progression demand radical proteomic reprogramming of cells concomitant with a modulation of the codon usage by the translational machinery. Accumulation of evidence advocates for acknowledgement of a primary role for hnRNP E1 in cell plasticity, and this is reinforced by identification of mechanisms involving hnRNP E1 that directly or indirectly converge upon the translational control of plasticity-associated proteins. We therefore propose that hnRNP E1, together with its associated proteins, acts as a hub that orchestrates the demands of proteome reprogramming during health and disease-associated cell plasticity.
